# Clinical guidelines for the management of depression with specific comorbid psychiatric conditions French recommendations from experts (the French Association for Biological Psychiatry and Neuropsychopharmacology and the fondation FondaMental)

**DOI:** 10.1186/s12888-019-2025-7

**Published:** 2019-01-30

**Authors:** D. Bennabi, A. Yrondi, T. Charpeaud, J.-B. Genty, S. Destouches, S. Lancrenon, N. Allaili, F. Bellivier, T. Bougerol, V. Camus, O. Doumy, J.-M. Dorey, F. Haesebaert, J. Holtzmann, C. Lançon, M. Lefebvre, F. Moliere, I. Nieto, C. Rabu, R. Richieri, L. Schmitt, F. Stephan, G. Vaiva, M. Walter, M. Leboyer, W. El-Hage, B. Aouizerate, E. Haffen, P.-M. Llorca, P. Courtet

**Affiliations:** 1 Service de Psychiatrie clinique, Centre Expert Dépression Résistante FondaMental, Centre Investigation Clinique 1431-INSERM, EA 481 Neurosciences, Université de Bourgogne Franche Comté, 25030 Besançon, France; 2 Service de Psychiatrie et de Psychologie Médicale de l’adulte, Centre Expert Dépression Résistante FondaMental, CHRU de Toulouse, Hospital Purpan, ToNIC, Toulouse NeuroImaging Center, Université de Toulouse, Inserm, UPS, Toulouse, France; 3 Service de Psychiatrie de l’adulte B, Centre Expert Dépression Résistante FondaMental, CHU de Clermont-Ferrand, Clermont-Ferrand, France; 4 SYLIA-STAT, 10, boulevard du Maréchal-Joffre, 92340 Bourg-la-Reine, France; 5 Service de Psychiatrie adulte, Centre Expert Dépression Résistante FondaMental, Hôpital Fernand-Widal, Paris, France; 6 Service de Psychiatrie de l’adulte, CS 10217, Centre Expert Dépression Résistante FondaMental, CHU de Grenoble, Hôpital Nord, Grenoble, France; 7 Clinique Psychiatrique Universitaire, Centre Expert Dépression Résistante FondaMental, CHRU de Tours, Université de Tours, Inserm U1253 imaging and Brain: iBrain, Tours, France; 8 Pôle de Psychiatrie Générale et Universitaire, Centre Expert Dépression Résistante FondaMental, CH Charles Perrens, UMR INRA 1286, NutriNeuro, Université de Bordeaux, Bordeaux, France; 9 Old Age Psychiatry Unit, pôle EST, Centre Hospitalier le Vinatier, Bron, France; 10 Brain Dynamics and Cognition, Lyon Neuroscience Research Center, INSERM U1028, CNRS UMR 5292, Lyon, France; 11 Geriatrics Unit, CM2R, Hospices civils de Lyon, Hôpital des Charpennes, Villeurbanne, France; 12 Service universitaire des pathologies psychiatriques résistantes, Centre expert FondaMental, PSYR2 Team, Lyon Neuroscience Research Center, INSERM U1028, CNRS UMR5292, Centre Hospitalier Le Vinatier, University Lyon 1, Bron, France; 13 Pôle Psychiatrie, Centre Expert Dépression Résistante FondaMental, CHU La Conception, Marseille, France; 14 Département des Urgences et Post-Urgences Psychiatriques, Centre Expert Dépression Résistante FondaMental, CHU Montpellier, Univ Montpellier, Montpellier, France; 15 DHU PePSY, Pole de psychiatrie et d’addictologie des Hôpitaux Universitaires Henri Mondor, Université Paris Est Créteil, Créteil, France; 16 Service hospitalo-universitaire de psychiatrie d’adultes et de psychiatrie de liaison - secteur 1, Centre Expert Dépression Résistante Fondamental, CHRU Brest, hôpital de Bohars, Bohars, France; 17 Service de Psychiatrie adulte, Centre Expert Dépression Résistante FondaMental, CHU de Lille, Hôpital Fontan 1, Lille, France

**Keywords:** Major depressive disorder, Anxiety disorders, Substance use disorders, Elderly, Pharmacotherapy

## Abstract

**Background:**

Recommendations for pharmacological treatments of major depression with specific comorbid psychiatric conditions are lacking.

**Method:**

The French Association for Biological Psychiatry and Neuropsychopharmacology and the fondation FondaMental developed expert consensus guidelines for the management of depression based on the RAND/UCLA Appropriatneness Method. Recommendations for lines of treatment are provided by the scientific committee after data analysis and interpretation of the results of a survey of 36 psychiatrist experts in the field of major depression and its treatments.

**Results:**

The expert guidelines combine scientific evidence and expert clinician’s opinion to produce recommendations for major depression with comorbid anxiety disorders, personality disorders or substance use disorders and in geriatric depression.

**Conclusion:**

These guidelines provide direction addressing common clinical dilemmas that arise in the pharmacologic treatment of major depression with comorbid psychiatric conditions.

## Background

Major depressive disoder (MDD) is becoming a major challenge for public health and medical practice, directly accounting for 4.4% of disease burden worldwide [1]. MDD is one of the most frequently recurrent psychiatric disorders, associated with a high risk of chronicity. Depressive disorders can occur comorbidly with nearly every other form of psychopathology in an unsystematic way, leading to greater symptom severity, disability, more suicides and poorer treatment response [2, 3]. Studies of adults patients with MDD reveal high prevalence rates of current social anxiety disorder (25,6%), panic disorder (11,1%), generalized anxiety disorder (20,8%), obsessive-compulsive disorder (13,4%), post-traumatic stress disorder (18,8%), alcohol abuse/dependance (11,9%) and drug abuse/dependance (7,3%) [4]. Despite advances in patient’s management, response rates to first-line antidepressants are moderate (40–60%), and remission is achieved in a minority of patients. For instance, in the STARD study, the remission rate reaches up to 60% after four subsequent trials although the probability of remission drops significantly after the failure of two consecutive antidepressants [5, 6]. Given the public health consequences of inadequately treated depression, guidance regarding treatment selection for MDD could have major impact. A number of guidelines have already been developed by professional societies with the aim to standardize treatments and to orientate everyday clinical practice towards evidence base. They integrate recommendations draws up after a critical analysis of available data, which are selected and ranked according to their level of evidence. Each compound is assigned a Level or Category of evidence (CE), which basically describes the level of efficacy. Subsequently, different Clinical recommendations or Recommendation Grades (RG) are implemented, which integrate additional clinical aspects of safety, tolerability and effectiveness. Despite a rigorous approach at both scientific and methodological levels, Evidence-Based Guidelines (EBG) can be limited in their usefulness and applicability by a number of different factors, including the availability of high-quality research evidence, the generalisability of research findings and the uniqueness of individuals with depression. One of the main obstacle stems from the use of restrictive criteria in most high-level Randomized Controlled Trials (RCT) excluding psychiatric comorbidities and elderly patients. Although the issue of comorbidity is recognized by EBGs, few specific recommendations are provided regarding acute treatment with antidepressant drugs, practical issues in prescribing and management or next-step treatment in those complex clinical situations. In these cases, the Formal Consensus method, based on the practical experience of a panel of experts, offer support strategies and appears to be particularly relevant. This method has been previously used by the French Association for Biological Psychiatry and Neuropsychopharmacology (AFPBN) and the fondation FondaMental (www.fondation-fondamental.org) to make recommendations for pharmacological and psychological strategies in depression (Bennabi et al., submitted).

The French National Health agency recommends the Formal Consensus method when the following conditions are met:

- No or insufficient level of evidence addressing the question.
- Possibility to decline the topic in easily identifiable clinical situations.
- Need to identify and select the strategies deemed appropriate by an independent panel from amongst several alternative options.

The AFPBN and the fondation FondaMental have developed a series of recommendations expected to provide a clear guidance regarding treatment options for specific populations/situations such as psychiatric comorbid disorders (ie anxiety disorders, personality disorders and addictions) and elderly, complementary to the Formal Consensus Guidelines for treating adults with treatment-resistant depression (TRD). We provide a synthesis of the deliberations of a panel of experts highly specialized in the field of unipolar depression, thereby enabling to establish recommendations for the pharmacologic treatment of MDD in elderly patients, patients with comorbid anxiety disorders, personality disorders or substance use disorders.

## Methods

The methodology has been previously described (see recommendations for treating TRD (Bennabi et al., submitted)). Recommendations were determined using the RAND/UCLA Appropriateness Method. This method combines scientific evidence and expert opinion to assess the appropriateness of medical procedures. It involves the following steps: (I) comprehensive review and analysis of the literature with regard to the research question conducted by a scientific committee (II) elaboration of the questionnaire to collect experts’ opinions for several highly detailed and illustrative clinical presentations; (III) scoring of the questionnaires; (IV) analysis of the experts’ opinions and drafting of the final report; (V) Peer-review phase; (VI) diffusion of the recommendations. This consensus survey of expert opinion on the pharmacologic treatment of MDD in specific populations/situations (elderly and comorbidities with anxiety disorders, personality disorders or addictions) was undertaken by the French Association of Biological Psychiatry and fondation FondaMental. The written survey was completed by 36 psychiatrist experts in the field of major depression and its treatments.

The scoring of the questionnaires was made using a modified version of the RAND 9-point scale for rating the appropriateness of medical decisions. Each expert answered each question with a graduated scale from 0 to 9 derived from a variation of the “Nominal Group” method, developed by the Rand Corporation and the University of California in the USA (“RAND/UCLA appropriateness rating method”), 0 meaning a “total disagreement” or “a formal contraindication” and 9 indicating a “total agreement” or “a formal indication”. This process is meant to detect agreement among experts without trying to promote consensus and potentially reduce the real differences of clinical opinion. This method has been previously used by the AFBPN to make recommendations for the use and management of long-acting injectable antipsychotics in bipolar disorder and for the management of TRD [7].

## Recommendations

### Comorbid anxiety disorders

#### During the first episode of MDD with comorbid anxiety disorder, it is recommended in first intention

- The concurrent treatment of both disorders
- The disease management by the same therapist as much as possible, which includes systematically a cognitive behavioural therapy along with a closer follow-up.

Strategies recommended for the pharmacological treatment of MDD depending on the comorbid anxiety disorder are presented in Table 1.

**Table 1:** Therapeutic strategies depending on comorbid anxiety disorder

Comorbidity	First intention	Second intention	Contra-indications
Obsessive-compulsive disorders	- SSRI - SNRI - Psychotherapy in combination	- Imipraminic - α2 Antagonist - Association of two ATDs from different pharmacological class - Potentiation with AAP	- Tianeptine - Irreversible non selective MAOI - First-generation antipsychotic - Anticonvulsant - Bupropion
Panic disorders		- Imipraminic - α2 Antagonist	- Association of ATDs - Anticonvulsant - First-generation antipsychotic - AAP
Social anxiety			
Generalised anxiety disorder			
Post-traumatic stress disorder			

*AAP* Atypical antipsychotic, *ATD* Antidepressant, *MAOI* monoamine oxidase inhibitor, *SSRI* Selective serotonin reuptake inhibitors; SNRI: Dual serotonin and norepinephrine reuptake inhibitors

### Comorbid substance use disorders

#### During the first episode of MDD with comorbid substance use disorders, it is recommended in first intention

- The recourse to full time hospitalization or
- The close monitoring in consultation (at least weekly),
- The electrocardiogram before treatment administration,
- The initiation of a substitution treatment in opioid drug dependence

More specifically, in case of severe alcohol addiction, the following procedure must also be considered in first intention, including:

- The close biological monitoring (complete blood count, blood electrolyte, liver and renal functions),
- The treatment of physical withdrawal syndrome,
- The prescription of antidepressant treatment after reassessment of mood, once appropriate care for physical withdrawal syndrome is over.

Strategies recommended for the treatment of MDD depending on the comorbid substance use disorder are presented in Table 2.

**Table 2:** Therapeutic strategies depending on comorbid substance use disorders

Comorbidity	First intention	Second intention	Contra-indications
Severe substance use disorders *(except alcohol and nicotine)*	- SSRI - SNRI - α2 antagonist - Concurrent treatment of MDD and addiction - Disease management involving a team specialised in addictology, psychoeducational groups or a psychotherapy focusing on addictive relapse prevention	- Imipraminic - Agomelatine	- Tianeptine - Irreversible non selective MAOI - First-generation antipsychotic - Association of ATD - Anticonvulsant
*Severe comorbid alcohol addiction*	- SSRI - SNRI - α2 antagonist - Structured Psychotherapy		- Disulfiram - Tianeptine - Bupropion - Irreversible non selective MAOI - Treatment of MDD alone
*Active smoking*	- SSRI - SNRI - α2 antagonist - Structured Psychotherapy - Concurrent treatment of MDD and smoking-cessation	- Imipraminic - Agomelatine	- Irreversible non selective MAOI - Tianeptine

*AAP* Atypical antipsychotic, *ATD* Antidepressant, *MAOI* monoamine oxidase inhibitor, *MDD* Major depressive disorder, *SSRI* Selective serotonin reuptake inhibitors, *SNRI* Dual serotonin and norepinephrine reuptake inhibitors

### Personality disorders

#### During the first episode of MDD with comorbid personality disorder, it is recommended

- The use of SSRI or SNRI in monotherapy or in combination with a psychotherapy in first intention
- The prescription of imipraminic antidepressant or α2 antagonist in second intention

Second-generation Antipsychotic or Acid valproic derivatives in association with the ongoing antidepressant treatment could possibly be considered in second intention.

Brain stimulation techniques are fully non-recommended.

### Geriatric depressive disorder

#### During an episode of MDD in adults over 65 years of age, it is recommended in first intention to perform a physical examination and ordering laboratory investigations to identify any medical problems that could contribute to or mimic depressive symptoms

- A clinical examination
- A Biological check-up (ie Complete blood count, blood electrolyte, liver and renal functions, Thyroid-Stimulating Hormon)
- An Electrocardiogram
- A Mini Mental State Examination
- The Assessment of the severity of patients’ clinical condition with clinician-rated and self-rated scales.

According to the experts’ panel, MRI may be considered in those with in late or very late onset first episode depression, those having associated neurological signs and those experiencing treatment resistant depression.

Anxiolytic treatment is not recommended in association with the current antidepressant. If used, hydroxyzine as well as benzodiazepines with short half-life must be preferred (for instance oxazepam).

Therapeutic strategies for MDD in patients over 65 years of age depending on clinical features of the episode are presented in Table 3.

**Table 3:** Therapeutic strategies in geriatric depression

Clinical features	First intention	Second intention	Contra-indications
Mild to moderate intensity	- SSRI - α2 antagonist	- SNRI - Agomelatine	- Irreversible MAOI - Bupropion - Association with an ATD from the same pharmacological class - Anticonvulsant - ECT
Moderate to severe intensity	- SSRI - SNRI - α2 antagonist	- Imipraminic	- Bupropion - Association with an ATD from the same pharmacological class - Anticonvulsant - First generation antipsychotic
Severe cognitive impairments	- SSRI - SNRI	- α2-antagonist - Agomelatine	
Severe psychomotor agitation	- SSRI - α2 antagonist	- SNRI - Potentiation with AAP	- Bupropion - Tianeptine - Irreversible MAOI - Association with an ATD from the same pharmacological class
Severe psychomotor retardation	- SSRI - SNRI	- α2-antagonist - Imipraminic - ECT in association	- Tianeptine - Bupropion - Association with an ATD from the same pharmacological class - First generation antipsychotic
Severe sleep disorders	- SSRI - α2 antagonist	- SNRI - Agomelatine	- Tianeptine - Irreversible MAOI - Bupropion - Association with an ATD from the same pharmacological class
Severe anhedonia	- SSRI - SNRI	- α2-antagonist - Imipraminic - Agomelatine	- Association with an ATD from the same pharmacological class - Anticonvulsant - First generation antipsychotic
Psychotic symptoms	- SNRI - Potentiation with AAP	- SSRI - α2-antagonist - Imipraminic - ECT in association	- Tianeptine - Irreversible MAOI - Bupropion - Association with an ATD from the same pharmacological class
High suicidal risk	- SSRI - SNRI	- α2-antagonist - Imipraminic - ECT in association Potentiation with AAP	- Tianeptine - Bupropion - Association with an ATD from the same pharmacological class - First generation antipsychotic

*AAP* Atypical antipsychotic, *ATD* Antidepressant, *ECT* Electroconvulsive therapy, *MAOI* monoamine oxidase inhibitor, *SSRI* Selective serotonin reuptake inhibitors, *SNRI* Dual serotonin and norepinephrine reuptake inhibitors

## Discussion

The main interest of the current expert guideline is to provide insights into the treatment practices of clinician experts across a number of common and complex clinical situations, with a particular attention paid to first and second-line strategies in patients with comorbid depressive disorders and anxiety, substance abuse or personality disorders and in elderly patients. Although the issue of comorbidity is recognized by EBGs, few specific recommendations are generated to aid clinician decision making at different steps of treatment, especially following the failure of the first-line strategy. This probably reflects the paucity of extant primary studies addressing these critical clinical questions. In such instances, CBG methodologies help to fill the gap between empirical literature and clinical practice and to reduce disparities in care. While some differences between EBG and CBG can be mentioned, the combination of these two approaches contributes to significantly facilitate and guide the treatment decision and choice in routine clinical practice.

### Recommendations for comorbid depression and anxiety disorders

The treatment of comorbid anxiety and depression requires specific psychopharmacological adjustments as compared to treating either condition alone. EBGs encouraged to screen and systematically monitor for comorbid anxious conditions in all individuals with mood disorders, although it is often unclear whether a sequential or a concurrent approach should be preferred for their management [8–11]. The UK National Institute for Health and Clinical Excellence (NICE) guideline directly acknowledged this shortcoming, and proposed a sequential approach that targets “the primary disorder” first (ie the one that is more severe and in which it is more likely that treatment will improve overall functioning) [12]. Experts’support for using Selective serotonin reuptake inhibitors (SSRI) or Dual serotonin and norepinephrine reuptake inhibitors (SNRI) as first-line treatment in depression comorbid with a spectrum of anxiety disorders is consistent with an extensive literature documenting safety and efficacy of these agents for both coexisting disorders [13]. Some but not all research have suggested that dual action drugs such as SNRIs might be superior to SSRIs for treating simultaneously both disorders [14]. Second-intention pharmacological approaches include trials of alternative medications as monotherapies with an Imipraminic or an α2 antagonist. Tricyclic antidepressants (TCA) cover both depression and certain anxiety disorders including GAD or PD, but are less commonly used due to tolerability issues [15–17]. Clomipramine appeared to be more effective than placebo in the treatment of Obsessive-compulsive disorders (OCD), and is recommended by the World Federation of Societies of Biological Psychiatry (WFSBP) and the American Psychiatric association (APA) in this indication [8, 9]. Alpha2 antagonists are also considered by the experts as second-line treatment, consistent with findings from randomized trials demonstrating mirtazapine’s anxiolytic and overall efficacy in MDD with prominent anxiety symptoms [18]. Notably, mirtazapine might be especially effective in patients with MDD and comorbid GAD, SAD or PTSD [19–21]. For the pharmacological management of MDD with comorbid OCD, the expert’s panel also favoured using adjunctive AAP or an association of two ATDs from different pharmacological class in second line. These special caution on OCD probably reflect difficulties in their management, as OCD have a reduced overall pharmacological response to ATD compared to GAD, panic disorder, PTSD and SAD [22]. However, there is currently no convincing evidence for the efficacy of a combination of antidepressants in this specific indication, except for the combination of SSRIs with clomipramine [23, 24]. Regarding add-on strategies, risperidone is among the most recommended treatment options for augmenting SSRIs in OCD while quetiapine and aripiprazole have both demonstrated efficacy as adjuncts for MDD with comorbid anxiety disorders in one RCT and three open label trial [25–29].

The expert recommended a concomitant use of ATD and psychotherapy (PT) in first intention, while some EBGs for the treatment of depression and comorbid anxiety disorders, for example, that of the WFSBP, the APA, the Canadian Network for Mood and Anxiety Treatments (CANMAT) propose to consider PT in combination or as an alternative to pharmacotherapy in case of non- or partial response, and frequently cited Cognitive Behavioral Therapy and Interpersonnal Therapy [8–10]. To date, it has not been clearly established whether combined treatments with psychotherapy and antidepressant medication lead to higher effects than the sum of the two treatments alone. Moreover, from a clinical point of view, the lack of available psychological treatments limits their use. In such instances, self-guided internet-based cognitive behavioral therapy could be a promising alternative due to its potential to increase access and availability of evidence-based therapy [30].

### Recommendations for comorbid depression and substance abuse/dependance

Experts’ support for treating co-occurring depression when it persists after at least a brief period of abstinence from substance use is consistent with several EBGs, even though most of them do not identified a preferred pharmacological option [8–11]. SSRI and SNRI are considered as an attractive first-line treatment among substance-dependent patients, probably because of their safety profile, and minimal sedating effects. If an SSRI or SNRI trial fails, consideration should be given to an imipraminic agent or agomelatine. A 2004 meta-analysis by Nunes and Levin found evidence for a modest beneficial effect of ATD on mood symptoms in depressed subjects with SUDs [31]. However, it should be notice that safety concerns regarding TCA have impeded both research and clinical use of these drugs in depressed patients with comorbid SUDs [32].

Regarding treatment options for patients with comorbid alcohol dependence, beside SSRI and SNRI, the use of an α2 antagonist in first line reflects the current empirical literature providing some evidence of efficacy for mirtazapine to elevate mood in persons with co-occurring MDD/ alcohol use disorders, without effectiveness for decreasing the level of alcohol ingestion [33]. Some guidelines, for exemple that of the CANMAT provides a particular sequence of interventions with first, second- and third-choice therapies, with several options within each intervention step [10]. In the specific case of smoking cessation, only the APA provided specific recommendations and proposed to consider bupropion or nortriptyline based on evidences suggesting that these agents provides an additional long-term benefit [9]. Experts’ recommendations were limited to SSRI, SNRI and α2 antagonists in first intention, however it should be noted that bupropion has not yet received approval for MDD in France.

Globally, it should be critically considered that data are sparse, in part because individuals with SUDs are traditionally excluded from medication trials for MDD and trials in dependant patients suffers from several methodological limits, including short trials lengths, inadequate ATD doses, and selection of patients with cross-sectional measures of depressive symptoms.

### Recommendations for comorbid depression and personality disorders

Key-issues identified with regards to the overlap of major depression and personality disorders include whether personality traits or disorders modify treatment responsiveness and outcome of mood disorder [34]. Due to the scarcity of original treatment studies specifically focused on these conditions, most EBGs provide general guidance but do not include hierarchical levels of evidence for pharmacotherapy. The CANMAT emphazised that patients with mood disorders and concurrent personality disorders should be treated with a combination of diagnostic-specific pharmacotherapy and PT, but without specified if a concurrent or a sequential approach should be favoured, while the APA adviced targeting the depressive disorder [9, 10]. The recommandations from the expert’s panel, and the APA are consistent, with SSRI as a first-line strategy, alone or in combination with PT [9]. The advice on pharmacotherapy was also similar on the use of low-dose antipsychotics and some antiepileptic medications for treating impulsivity and loss of control.

### Recommendations for geriatric depressive disorders

Most of the European and North American EBGs have no special chapter or separate section for geriatric depression. General principles for treating older adults are similar to those described for younger adults, with special caution for adverse drug reactions, potential drug interactions and somatic comorbidities [12]. Moreover, EBGs acknowledged that older patients would usually require lower doses of medication, due to the association of aging with reductions in renal clearance and volume of distribution and concomitant medications. The formalized consensus of our experts’ panel provides recommendations on older adults with depression based on several dimensional features. Overall, experts’ support for using SSRI or SNRI as first-line treatment is congruent with the CANMAT, the APA and the WFSBP guidelines, documenting both safety and efficacy of these strategies [8–10, 35]. The CANMAT provides a detailed stepped approach with a first, a second and a third-line intervention [10]. The use of α2 antagonists in MDD with severe sleep disorders or psychomotor agitation adviced by the experts probably results from its sedative properties and its more rapid onset of action. This assumption in mainly based on the study by Schatzberg and colleagues showing that mirtazapine exhibit more notable antidepressant effects than the standard SSRI paroxetine, with greater reductions in anxiety levels, somatic symptoms and sleep disturbances in 255 elderly depressed patients [36]. Despite two RCT and one naturalistic trial supporting the use of bupropion in older adults with MDD, and while both the Canadian and the US guidelines recommend its use as first and second-line treatment, the experts we surveyed do not advocated its use, due to the absence of approval for the treatment of MDD in France [9, 10, 37].

The formal consensus method is used to specify a prescription framework for specific populations or clinical situations for which evidence are scarce or debated, which is the case in major depressive disorder with psychiatric comorbid disorders. Indeed, in those situations, empirical literature is lacking for specific iterative medications. However, a limited number of comorbid conditions were included in our study. Comorbid anxiety and substance use disorders are frequently associated with MDD, but there is also substantial overlap with other medical and psychiatric comorbidities (e.g. attention deficit hyperactivity disorder) for whom a thorough investigation is needed. Second, expert’s prescribing choice may be influenced by perceived therapeutic actions or perceived risk of adverse drug events, or both leading to provide recommendations even when little evidence of superior efficacy of one agent over another exists in the literature [38, 39].

## Conclusion

Within the limits of expert opinion and with the expectation that future research data will take precedence, these guidelines provide direction for addressing common clinical dilemmas that arise in the pharmacological treatment of MDD with comorbid conditions.

Although standard guidelines contribute to facilitate and guide the treatment decision and choice for the clinicians, they cannot take into account the overall complexities involved in the care of each individual patient or substitute professional knowledge and clinical judgement. Importantly, additionnal researches concerning the temporal onset of the disorders, similarities and differences in neurobiological substrates, heritability, and environnemental stressors associated with each disorder are needed. Identification of shared etiological factors that underlies the comorbidity in depressive disorders may contribute to the development of better interventions that target not only MDD but also other major sources of psychiatric morbidity. Adequately powered, well-controlled studies are further needed to show the effectiveness of treatments for depression with comorbid under conditions of routine clinical practice.

## Abbreviations

AAP: Atypical Antipsychotic
AFBPN: Association for Biological Psychiatry and Neuropsychopharmacology
EBG: Evidence-based guidelines
ECT: Electroconvulsive therapy
GAD: Generalised anxiety disorder
MAO: Monoamine oxidase inhibitors
MDD: Major depressive disorder
OCD: Obsessive-compulsive disorders
PD: Panic disorders
PTSD: Post-traumatic stress disorder
RCT: Randomized controlled trials
SA: Social anxiety
SNRI: Dual serotonin and norepinephrine reuptake inhibitors
SSRI: Selective serotonin reuptake inhibitors
TCA: Tricyclic antidepressant
TRD: Treatment-resistant depression
